# Factors Affecting Exclusive Breastfeeding among Women in Muheza District Tanga Northeastern Tanzania: A Mixed Method Community Based Study

**DOI:** 10.1007/s10995-015-1805-z

**Published:** 2015-08-04

**Authors:** Aubrey R. Maonga, Michael J. Mahande, Damian J. Damian, Sia E. Msuya

**Affiliations:** St Augustine Muheza, Institute of Health and Allied Sciences, P O Box 5, Muheza, Tanga, Tanzania; Department of Community Health, Institute of Public Health, Kilimanjaro Christian Medical University College (KCMU Co), P O Box 2240, Moshi, Tanzania; Department of Epidemiology and Biostatistics, Institute of Public Health, Kilimanjaro Christian Medical University College (KCMU Co), P O Box 2240, Moshi, Tanzania; Department of Community Medicine, Kilimanjaro Christian Medical Centre, P O Box 3010, Moshi, Tanzania

**Keywords:** Exclusive breastfeeding, Factors, Women, Community based study, Tanzania, Worldwide, Sub Saharan Africa

## Abstract

**Objectives:**

Estimates shows exclusive breastfeeding (EBF) has the potential to prevent 11.6 % of all under-five deaths in developing countries. Prevalence of EBF is low globally (35 %), and in sub Saharan Africa ranges between 22 and 33 %. Like other developing countries the prevalence of EBF is 50 % in Tanzania. There is limited information in Tanzania on factors influencing EBF apart from information specific for HIV positive women. This study aimed at examining factors that affect EBF practice among women in Muheza district, Tanga region, northeastern Tanzania.

**Methods:**

A community based cross-sectional study using both qualitative and quantitative methods was conducted from April to June 2014. To collect relevant information, a total of 316 women with infants aged 6–12 months were interviewed using a questionnaire and 12 key informants using in-depth interview guide. Qualitative data was analyzed using thematic analysis while bivariate and multivariate logistic regression analysis were used assess association between EBF and predictor variables.

**Results:**

The prevalence of EBF was 24.1 %. The perception that mothers’ breast milk is insufficient for child’s growth, child being thirsty and the need to introduce herbal medicine for cultural purposes were among the important factors for early mixed feeding. In multivariate analysis advanced maternal age (OR 2.6; 95 % CI 1.18–5.59) and knowledge on EBF duration and advantages (OR 2.2; 95 % CI 1.2–3.8) remained significantly associated with EBF practice.

**Conclusions:**

The prevalence of EBF in our study is low compared with the national prevalence. Strategies to target beliefs that breast milk is insufficient for growth need to be strengthened in the community. Furthermore opportunity to increase EBF training during ante and postnatal visits for women should be enhanced as more than 90 % of women in the district use skilled attendants during pregnancy and delivery.

## Significance

In this community older women (35–49 years) and those with good knowledge of EBF had higher odds of exclusive breastfeeding their infants to others.

## Introduction

Exclusive breastfeeding (EBF), which is defined as giving an infant only breast milk from birth up to 6 months of age, without giving other liquids or solids, not even water, with the exception of oral rehydration solution, or drops/syrups of vitamins, minerals or medicines has been shown to be one of the evidence-based interventions for child survival [1, 2]. Estimates show that, good breastfeeding practices especially EBF could prevent about 11.6 % of the 6.9 million under five deaths in developing countries [2, 3].

EBF promotes optimal neonate and infant growth as it contributes to 100 % of daily nutrition requirement of children up to 6 months of age, 50 % of children of 6–12 months and 35 % of nutritional requirement for children aged 12–24 months [4]. EBF has also been shown to reduce neonatal and child deaths associated with diarrhea and acute respiratory tract infections, two of the leading causes of child deaths [3, 5–8]. EBF contributes in reducing the risk of mother to child transmission of HIV [5, 9, 10]. This is a vital advantage in Africa where the prevalence of HIV infection is high and replacement feeding that is acceptable, feasible, affordable, sustainable and safe (AFASS) is unavailable for many HIV positive women [9–11].

Several strategies have been initiated by UNICEF and WHO in order to promote optimal breastfeeding practices i.e. start breastfeeding within 1 hour of birth, EBF for first 6 months of infant life and after 6 months introduction of appropriate weaning foods while continuing to breastfeed for 2 years [1–3]. Tanzania government has endorsed these global commitments to improve EBF practices. These strategies are Baby Friendly Hospital initiative (BFHI), Infant Young Child Feeding policy (IYCF) and breastfeeding recommendation in prevention of mother to child transmission of HIV (PMTCT) [1, 11–13].

Despite the benefits and efforts to promote breastfeeding, EBF is sub optimally practiced in many developing countries. Only 35 % of the infants are exclusively breastfed worldwide [14]. In sub Saharan Africa which has high rates of infants and child mortality only 33 % of infants are exclusively breastfed [3, 14]. The EBF coverage of 90 % is required in order to benefit from 11.6 % reduction of child death in low income countries [2, 15]. Tanzania’s goal is to have EBF coverage of 80 % by 2015, but the prevalence of EBF is still low; was 43 % in 2004 and slightly increased to 50 % by 2010 [12, 13, 16].

Several factors have been found to be associated with EBF, in developed countries; social class, level of education, age of the mother, lack of parental support, living with partner, employment status, parity, place of delivery, smoking during pregnancy and presence of BFHI policies [17, 18]. Similarly in developing countries, socio demographic factors like maternal age, education, employment, residency, cultural and religious practices, living arrangement, antenatal care practices, home delivery, professional assistance at birth were associated with suboptimal breastfeeding practices [19–21, 25, 29, 31]. But the way the factors influence EBF practice differ in direction from one setting to the other, necessitating the need for setting specific data.

Despite the low prevalence, few studies have been done in Tanzania regarding factors that influence EBF practice. Some were done before implementation of IYCF or PMTCT policy to promote and protect EBF [21, 22] and others were done among the HIV positive women population only [23, 24]. Two recent quantitative studies have both shown that 50–80 % of the women are aware that infants should be exclusively breastfed up to 6 months, yet majority of women mix fed early [25, 26]. The current study went a step further and used both qualitative and quantitative methods to explore in addition to factors influencing EBF, it also assessed women’s and community beliefs and norms that affects breastfeeding practices despite seemingly high knowledge and awareness of the EBF [15].

## Methods

### Design and Site

A community based cross-sectional study was conducted in April and June 2014 at Muheza district using both quantitative and qualitative methods. Muheza is one among eight districts of Tanga region situated at north-eastern Tanzania. It has a population of 204,461, of whom 45,008 are women of reproductive age and 6265 infants [27]. The district is divided administratively into four divisions namely Muheza, Ngomeni, Bwembwera and Amani and 33 wards and 522 hamlets. The large population of Muheza district depends on agriculture and livestock keeping. Other economic activities are fishing and small petty business. Few people are employed by the public sector. The district has 47 health facilities; one district hospital, three health centres and forty-three dispensaries among these 43 provide reproductive and child health services [28].

### Study Population

The study enrolled women with infants aged 6–12 months at Muheza. Women were excluded if they had never breastfed the infant or if had an infant who experienced severe neonatal problems that necessitated admission into neonatal unit and interfered with breastfeeding in the first days of life.

For qualitative component of the study in addition to the women, the study also enrolled male partners, mother in laws and traditional birth attendants (TBAs).

### Sample Size and Sampling

The formula for precision by Leslie Kish was used to calculate the sample size. The alpha was set at 5 %, power of 80 % and EBF prevalence of 20.7 % was used and minimum sample required was 278. For qualitative part of the study, the sample size depended on saturation point.

Multi-stage sampling technique was used to select women with infants aged 6–12 months. First stage involved purposive selection of two divisions out of four, the two divisions were selected one to represent an urban setting and another rural setting. In the second stage two wards were randomly selected from each division making a total of four wards. Then five villages\hamlets were randomly selected from each ward making a total of 20 villages/hamlets. Thereafter, a list of women of reproductive age (WRA) eligible to participate in the study was made with the help of village/hamlet leaders. A total of 326 women met the inclusion criteria and were invited participate in the study.

For qualitative component one woman with her partner and mother in law was purposively selected per ward. TBAs were selected from two divisions as advised by district reproductive health coordinator.

### Study Procedures

Before enrollment, the study purpose was explained to participants and those who agreed to participate in the study gave a written consent. Participants who were unable to write, right thumb was regarded as a signature. Questionnaires were used to collect quantitative data and in-depth interview guide were used to collect qualitative data.

Face to face interviews were conducted at participant’s home, at private area in order to understand each other and to observe confidentiality. Questionnaires were used to collect information from women and included socio-demographic characteristics (age, marital status, education, employment, alcohol intake), reproductive information (parity, place of delivery, ANC attendance, advice on breastfeeding during ANC and postnatal period), breastfeeding practices (time of initiation of breastfeeding, use of pre lacteals, time of introduction of water, liquid and solids and EBF practice) and knowledge on EBF duration and advantages.

In depth interviews were also conducted at homes using interview guides. Topics addressed were; relationship status, duration of breastfeeding, other types of food given (time started, how did she come to decide, partner and other people involved in the decision), perception on EBF, tradition that govern what to be given to the child after birth (its role and how it affects the child) and norms that suggest how to feed the child (if age specific and their custodians/gatekeepers).

### Data Analysis

Statistical package for social sciences (SPSS) version 16.0 was used for analysis of data and thematic analysis for qualitative data. Mothers who did not introduce liquids/semisolids to their infants up to 6 months were categorized as practiced EBF. Knowledge was measured using two questions; understanding the duration of EBF which is up to 6 months and mentioning at least two advantages of EBF to the child. Participants who knew the correct duration and could mention at least two advantages of EBF were categorized as having good knowledge on EBF while those who could not mention/answer both questions were categorized as having poor knowledge. Descriptive statistics were used to summarize data. Measures of central tendency and dispersion were used to summarize numerical data, proportion (percentage) to summarize categorical variables. Association between dependent and independent variables was estimated in bi-variate and multivariate analysis. Odds ratio (OR) with 95 % CI were computed. Explanatory variables found to be statistically significant in the bi-variate analysis were entered into multiple logistic regression analysis to control for confounders. A *P* value of <0.05 was considered statistically significant.

Qualitative data were analyzed using thematic analysis where familiarization, coding, interpretation of the findings and summarizing major themes from findings was done. Key themes are presented with using of paraphrase and/or direct quotes to elaborate more.

### Ethical Approval

Permission to conduct this study was obtained from the KCMU College ethics research committee (Certificate number 665). Permission was sought from the District Medical Officer and leaders of selected areas. Written consent was sought from every participant. For participants who were unable to write, a right thumbprint was accepted as a signature.

## Results

### Background Characteristics of the Women and Infants

Out of 324 women who were approached and invited to participate, 316 agreed, giving a response rate of 97.5 %. A total of 12 participants participated in the qualitative part of the study. The mean age (standard deviation) of the 316 participants was 27.5 (SD = 6.1) years. The majority, 237 (75 %) of the participants were married or cohabiting, were not formally employed (93 %), had primary education (69 %), and more than a half (59 %) had <3 children, Table 1. Participants’ partners age ranged from 18 to 70 years with mean of 33.4 years (SD, ±7.5) and majority of the partners (69 %) had primary education. The median age of the infants was 9 months (range 6–12 months), and 53 % were males, Table 2.

**Table 1:** Socio-demographic characteristics of participants and their partners, (N = 316)

Variable	Total N (%)	EBF (n = 76)		*P* value
		n	%	
*Mothers’ information*				
Age in years^a^				
Age group (years)	27.5(6.1)			
15–24	129	24	18.6	0.034
25–34	142	35	24.6	
35–49	45	17	37.8	
Marital status				
Married	149	47	24.6	
Cohabiting	46	10	21.7	
Single/widow/divorced	79	19	24.1	0.920
Level of education				
None	33	6	18.2	0.674
Primary	218	53	24.3	
Secondary or higher	65	17	26.2	
Employed for cash				
No	296	66	22.4	0.012
Yes	20	10	50.0	
Alcohol intake				
No	299	72	24.1	0.990
Yes	17	4	23.5	
Number of living children				
1 child	114	27	24.1	0.987
2 children	72	17	23.6	
3 or more	130	32	24.6	
*Partner’s information*				
Age in years				
< or = 34	179	42	23.6	0.802
>34	137	34	24.8	
Partners level of education				
None/primary	222	49	22.1	0.206
Secondary or higher	94	27	28.7	

^a^Adjusted for mothers’ age, employment, counseling on EBF during pregnancy and postnatal period and composite/overall knowledge on EBF

**Table 2:** Child characteristics associated with EBF practice (n = 316)

Variable	Total N (%)	EBF (n = 76)		*P* value
		n	%	
Birth weight				
<2.5 kg (LBW)	21	6	28.6	0.629
2.5 or higher	295	70	23.9	
Birth order of current child				
First child	113	27	23.9	0.990
Second child	72	17	23.6	
Third child or higher	131	32	24.4	
Place of delivery current child				
Home delivery	28	4	14.8	0.251
Facility delivery	288	71	24.7	
Type of HF current child delivered (n = 288)				
Dispensary	27	6	22.2	0.781
Health centre	14	6	42.9	
Hospital	247	59	23.9	
Mode delivery current child				
Normal delivery	279	66	23.7	0.938
By operation (C/S)	37	9	24.3	
Problems immediate after birth				
No	290	66	24.0	0.920
Yes	26	9	23.1	
Sex				
Boys	166	43	25.9	0.471
Girls	150	33	22.0	

Regarding last pregnancy; 95 % of the 316 women reported having attended for antenatal care and 91 % reported to have delivered at the health facilities, Table 3. Despite high attendance, only 39 and 25 % of the women reported having received breastfeeding counseling during antenatal care attendance or after delivery respectively.

**Table 3:** Reproductive and Maternal Health characteristics among WRA with infants aged 6–12 months in Muheza district (N = 316)

Variable	Total N (%)	EBF (n = 76)		*P* value
		n	%	
Gravida				
Primegravida	107 (34.0)	27	23.9	0.990
Multigravida	208 (66.0)	49	23.6	
ANC attendance—pregnancy of last child				
No	16 (5.1)	3	18.8	
Yes	300 (94.9)	73	24.3	0.770
Frequency of ANC visits during pregnancy of last child				
None	16 (5.1)	3	18.8	
1–3 times	45 (14.2)	10	22.2	
4+ times	255 (80.7)	63	24.7	0.820
Place of delivery last pregnancy				
Home	28 (8.9)	4	14.8	
Hospital	288 (91.1)	72	24.7	0.251
Level of facility deliver last baby (n = 288)				
Dispensary	27 (9.4)	6	22.2	
Health Centre	14 (4.9)	6	42.9	
Hospital	247 (85.8)	59	23.9	0.781
HIV testing during pregnancy of last child				
No	15 (4.7)	6	42.9	
Yes	301 (95.3)	70	23.3	0.110
Advice on EBF during ANC attendance				
No	194 (61.4)	39	20.1	
Yes	122 (38.6)	37	30.3	0.038
Post natal counseling on EBF				
No	237 (75.0)	28	20.3	
Yes	79 (25.0)	48	35.4	0.006

### Breastfeeding Practices and EBF Prevalence

Of the 316 women, 65 % reported they initiated breastfeeding within 1 h after child’s birth, 96.2 % fed their newborns colostrum and 7.6 % gave pre-lacteal feeding, Table 4. Among 24 women who gave pre-lacteal feeding; the most common was local herbs “*mavumba*” (54 %), followed by cassava porridge “*uji wa bada*” (25.0 %), lukewarm water (16.7 %) and other items like sugar or Gripe water (4.3 %).

**Table 4:** Breastfeeding practices and knowledge among 316 women with infants aged 6–12 months in Muheza, Tanga

Characteristic	Total (n)	Exclusively breastfed infants (n = 76)		*P* value
		n	%	
Time of onset of breastfeeding (h)				
Within 1 h	205	43	21.0	0.209
>1–24 h	103	31	30.1	
>24 h	8	2	25.0	
Feed baby colostrum				
No	16	1	8.3	0.306
Yes	300	75	25.0	
Breast problems within 6 months				
No	286	70	24.0	0.896
Yes	26	6	23.1	
Knowledge on duration of EBF				
Not knowledgeable	133	17	12.8	<0.001
Knowledgeable	183	59	32.2	
Could mention advantage of EBF to the child				
No	210	60	28.6	0.008
Yes	106	16	15.5	
Overall knowledge (duration and advantage)				
Poor knowledge	169	27	16.0	<0.001
Good knowledge	147	49	33.3	

**Fig. 1:**
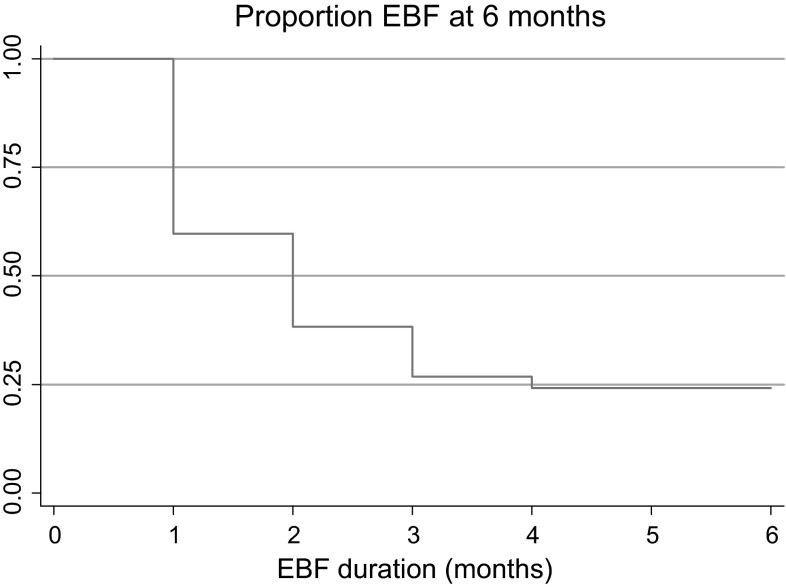
Exclusive breastfeeding by months among 316 women with infants aged 6–12 months Muheza, Tanga

The prevalence of EBF up to 6 months was 24.1 % (n = 76). While 76 % of the 316 participants exclusively breastfed their children up to 1 month after birth, this proportion dropped markedly to 24.1 % at 6 months, (Fig. 1).

**Fig. 2:**
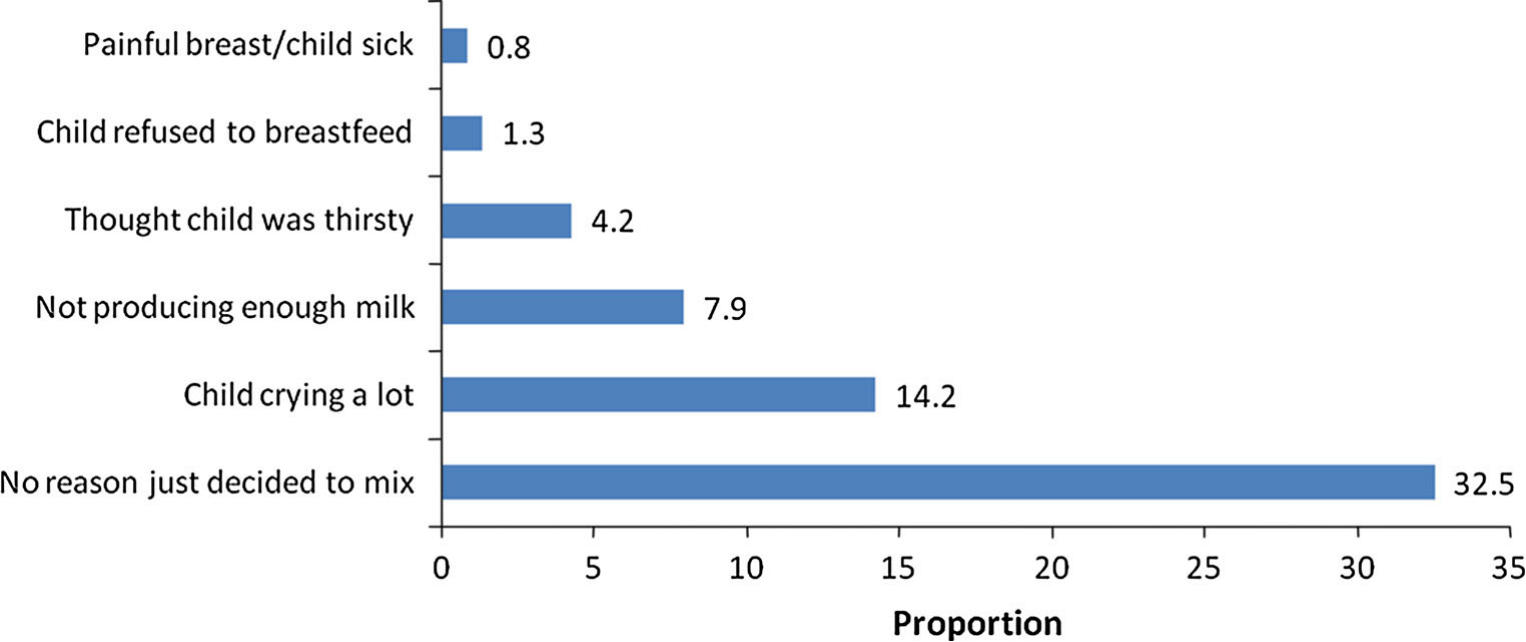
Reasons mentioned for mix-feeding before 4 months of age

Among those who mix-fed, common reasons mentioned for mix feeding before 4 months were; just thinking the child needs other foods (33 %), child was crying a lot (14 %) and not having enough milk (8 %), Fig. 2. Common supplementary foods included light cassava porridge and water.

Despite low prevalence of EBF, nearly 58 % (183) of the women were knowledgeable on the duration for EBF and more than two-thirds could mention two or more advantage of EBF, Table 4.

### Factors Influencing EBF Practice

The association between EBF and several factors is shown in Tables 1, 2, 3 and 4. Mothers’ age and employment for cash were significantly associated with EBF in bi-variate analysis. Employed mothers (50 %) exclusively breastfed more compared to others (22.4 %), *P* = 0.005, while older women aged 35+ (37.8 %) had higher prevalence of EBF compared to those aged 18–24 years (18.6 %), *P* = 0.03, Table 1. Infants characteristics like birth weight, birth order or sex were not associated with EBF, Table 2. Mothers who received advice on EBF during antenatal attendance had seventy-three percent higher odds of EBF compared to those who did not (OR 1.73; 95 % CI 1.03–2.92), similarly mothers who received counseling on EBF after delivery were two times more likely to practice EBF compared to those were not advised on EBF, (OR 2.21; 95 % CI 1.26–3.86), Table 3. Women with knowledge on breastfeeding duration (32.2 vs. 12.8 %, *P* < 0.001) and with knowledge on advantages of EBF (28.6 vs. 15.1 %, *P* = 0.008) had higher EBF prevalence compared to others, Table 4. In the logistic regression analysis mothers aged 35–49 and overall knowledge on EBF remained independently associated with EBF practice, (Table 5). Mothers aged 35 years or more had 2.6 higher odds to practice EBF compared to those age 15–24 years, while those with good knowledge on duration and advantages of EBF had 2.0 higher odds of exclusively breastfeeding compared to others.

**Table 5:** Logistic regression analysis of factors influencing EBF

Variable	Adjusted OR (95 % CI)	*P* value
Age group (years)		
15–24	1	
25–34	1.33 (0.72–2.45)	0.36
35–49	2.56 (1.18–5.59)	0.02
Employed for cash		
No	1	
Yes	2.35 (0.89–6.16)	0.08
Advice on breastfeeding during ANC attendance		
No	1	
Yes	1.22 (0.67–2.23)	0.53
Advice on breastfeeding during post natal period		
No	1	
Yes	1.71 (0.90–3.24)	0.10
Overall knowledge on exclusive breastfeeding		
Poor	1	
Good	2.15 (1.22–3.76)	0.008

### Results from In-Depth Interviews

A total of 14 informants participated, among them there were four men, four mothers in laws, two TBAs and four WRA. The age of informants ranged from 23 to 70 years, with the majority (71 %) being females. Key themes emerged that emerged were: mother’s milk not being enough for child growth, EBF was associated with HIV status, and the need to conform to some traditional practices after the baby was born.

All informants during KII expressed that breast milk is very important for the children, since it helps in nourishing their children. However, in spite the knowledge, majority could not breastfed their children exclusively for 6 months. Most of the informants strongly believed that mother’s milk alone for the first 6 months is not enough. They reported that on average children in the study area are exclusively breastfed for 3–4 months and it was uncommon to feed breast milk alone up to the age of 6 months.

My child did not reach six months ah ah did not do that, when he reached four months I started giving him porridge, and I used cassava porridge (uji wa bada) which was not too light and not too stiff, the mid state. The child will not grow and gain weight properly (WRA 1, aged 24).

I gave her water early and she started light porridge when was three months. When she reached about five months we made the porridge a bit stiff, made from millet. Eeeh we did this because breast milk was not enough and was too light so that she can get strength a little bit. (Mother in law 1, aged 58).

Majority of the informants explained reasons for mixed feed were mother’s milk not being enough which made the child to cry so much. Other reasons that were mentioned were; the baby needs other foods after 3 months to gain strength or attain good health, and a popular belief that one have to give light porridge for the baby to grow well.

But me I say it is not possible to give breast milk alone for six months, because six months is a lot for a human being, there must be something that will be given a little bit (Partner 2, aged 40).

You see when this baby was two months even after breastfeeding he continued crying, but when I started giving him cassava porridge he became calm. My milk was not enough to satisfy his hunger and this is usual at this age (WRA 2, aged 23).

I discussed with my daughter in law about introducing other foods to my grandson, since the child cried a lot after being breast fed. We thought breast milk was not enough, and as we gave him porridge he became calm, so that his mother can do other activities (Mother in law 2, aged 70).

You see I think breast milk of the mother is good, but it is not enough to make the baby grow well and gain weight. Our fellow tenant advised us to introduce thin cassava porridge at about three months and the baby really grew and gained weight, so I think cassava porridge after second or third month is good for the child (Partner 3, aged 50).

It was popularly believed that women who breastfed exclusively are HIV positive.

If the woman is HIV-positive I tell her she has to try not to mix for 3 months. Me, I don’t have a tablet which they are given at the clinic to reduce transmission, so she will have to try to feed the baby with her milk alone. For others I advice them to start cassava porridge when the baby cries too much as there is no harm like in those who are HIV-positive (TBA 1, aged 70).

If she has the virus (HIV), if is infected has to breastfeed for three months then stop and initiate the child on cow’s milk. (TBA 2, aged 65).

Here in this area people believe that if a woman does not introduce porridge early then she is HIV-positive. It is not normal at all to breastfeed for 6 months without mixing (Partner 4, aged 28).

They also believed some concoctions/local herbs or “*uji wa bada*” are needed when the child is born for cultural purposes and to relieve gastrointestinal upset. These herbs are given by certain old ladies or TBA. Other things that may be given are soil or sugar. This social norm was further believed to have no harm to the children.

Cassava porridge is good, child does not get abdominal colicky. Mhmh child does not get problems at all when you mix breast milk and cassava porridge (Mother in law 1, aged 58).

You know when a child is born he/she has to test the soil to signify he/she has arrived to the world. The child has then to be given sugar and vinegar to understand the world has a mixture of sweet and bitter things/experience. Nearly every family does that though they are not open (TBA 1, aged 70 years).

Mhh the child has to be given ‘mavumba’ or local herbs to prevent colicky pains. Every child gets pain, but if you give mavumba early you forget about abdominal problem (Mother in law 3, aged 51).

Others reported that they give water in small quantities before starting breastfeeding to stimulate sucking reflex.

You boil (water) put in a small bottle then give little amount of water using a small spoon so that a child can lick the spoon. Eeeh when the child does that it help to soften/open the chest, and stimulate sucking reflex (Mother in law 1, aged 58).

## Discussion

In this study it was found that prevalence of EBF among women of reproductive age with infants aged 6–12 months at Muheza was low (24.1 %) compared to the WHO recommended EBF coverage of 90 % and the national target of EBF coverage (80 %) [1, 14, 15]. Older mothers aged 35–49 years and women with good knowledge on EBF had higher prevalence of EBF practice. There was a strong belief that breast milk alone for the first 6 months of infant life is not enough for child growth.

Prevalence of EBF in this study is consistent with previous studies in Kilimanjaro (20.7 %) and in Uganda (24 %), but it was lower than the EBF prevalence shown by the Tanzania demographic health survey (TDHS) of 2010 (50 %) and for developing countries (35 %) [12, 13, 25, 29]. The difference of EBF observed between this study and TDHS may be due to methodologies used to estimate EBF. This study used recall since birth method while TDHS used 24 h recall. Using 24 h recall tends to overestimate the prevalence of EBF compared with recall from birth as shown by studies in Sweden, Uganda and Sri lanka [30–32].

Suboptimal breastfeeding practices were common; 35 % of the children were not breastfeed within 1 h, 8 % were given pre-lacteal feeding and 76 % were not exclusive breastfed. Nearly 21–51 % of the infants in Tanzania are not breastfed within 1 h after birth [12, 25, 26] while 14–31 % of the infants are given prelacteal feeding [12, 21, 22, 25, 26]. Recent review by Black et al. [2] showed that suboptimal breastfeeding results in nearly 800,000 child deaths annually in low income countries, or 11.6 % of death among children under 5 years of age [2]. Tanzania has made significant reductions in under five mortality from 157 per 1000 live births in 1990 to 54 per 1000 live births in 2013 [3, 15]. However neonatal mortality rate (NMR) of 21 per 1000 live births it is still off the target of NMR of 19 per 1000 live births to meet the MDG 4 goal by December 2015 [13], hence breastfeeding interventions especially early initiation of breastfeeding and EBF needs to be strengthened and scaled up. Thus there is a need to increase community awareness and education on advantages and negative effects of mix feeding [33].

Nearly 65 % of the women had given water by the 3rd month, 29 % had given porridge and 15 % had given cow’s milk. This is similar to the results by the TDHS of 2010 and researchers in Kilimanjaro which shows nearly 35 % of the infants <6 months had been given complementary feeding [12, 25]. Researchers in Uganda and Malawi have also shown complementary feeding is given to 25–50 % of the children before 6 months [29, 31, 34]. Appropriate complementary feeding is recommended by the WHO to start at 6 months of age, when breast milk is not sufficient to maintain child’s energy and nutrition requirements. Apart from mortality, lack of EBF has been associated with considerable morbidity. Mix fed children have higher prevalence of diarrhea and respiratory infections than EBF children [2, 6, 7, 14]. Further non-exclusive breastfed infants have been shown to have significantly higher rates of stunting compared to EBF children [2]. Stunting is a well-established risk factor for poor child motor and cognitive development among children. Cohort studies have shown stunting before age 2–3 years predicts poorer cognitive and educational outcomes in later childhood and adolescence [2, 35]. It seems policies to promote EBF like Baby Friendly Hospital initiative (BFHI) and infant and young child feeding (IYCF) of which the Tanzania government has endorsed are not optimally functioning and there is a need to ensure consistent implementation of these initiatives.

In this study good knowledge on EBF (i.e. required duration and advantages) was associated with twice the odds of EBF the infants. Nkala and Msuya [26] in Kigoma Tanzania also found that women with EBF knowledge were five times more likely to EBF compared to others, while other researchers did not find the association [12, 21, 25, 36]. Innovative strategies to increase women’s awareness and knowledge on breastfeeding in general and in EBF are needed outside the usual facility channel. One strategy that Tanzania may consider would be using EBF promotion peer counselors in the community or women’s groups which have recently been shown in community trials to increase the duration of EBF in African settings [37, 38].

It was noted that there was high antenatal attendance during previous pregnancy (95 %) and majority of the women delivered at health facility (91 %). However there were missed opportunities to counsel women on EBF as few women reported receiving advice on EBF during antenatal (39 %) and postpartum periods (25 %). In this study advice on EBF during pregnancy or immediate after delivery increased the prevalence of EBF in the bi-variate and not multivariate analysis. However results by the TDHS, from Kilimanjaro and from Kigoma all showed significant increase in the prevalence of EBF if the women are advised at facilities on EBF during these two critical time periods [12, 25, 26]. Health facilities and providers are trusted source of knowledge [22] and information and apart from increasing counseling efforts at facilities they should be leading in organizing linkage with community groups regarding breastfeeding as 63 % of the women reported that there is nobody providing advice on breastfeeding in the community.

Community beliefs influenced EBF in this setting. There were symbolic procedures done to the baby once he/she is born and people strongly believing mother’s milk alone is not enough for growth without giving porridge. The common reasons for early initiation of complementary food were inadequate breast milk, and mothers’ perception that the child was thirsty, these reasons given by women were observed in different settings as noted by other researchers in Tabora Tanzania, Malawi, Zambia, Nigeria and India [21, 29, 34, 39, 40]. There is a need to target this when designing public messages/communications in EBF.

The misconception that EBF is for HIV-positive women as perceived by male partners or TBAs is worrying. Male partners have been shown to be vital in facilitating achievement or adherence to several reproductive, maternal and child health interventions. Male support has been associated with use of skilled birth attendants, use of contraceptives, adherence to PMTCT intervention and increased use of vaccination services [41, 42]. Further if key gate keepers in the community like TBAs believe this, then the advice they would give will deter EBF interventions. The need for tailored interventions to target this misconception in Muheza is urgently needed.

Maternal age was significantly associated with EBF with young mothers having low EBF prevalence as was also found in other studies, however, these findings were contrary to others studies done which showed that maternal age was not associated with EBF practice [10, 33]. Therefore more effort should be targeted to young mothers during intervention to promote EBF. Other socio demographic characteristics like marital status, level of education and alcohol intake did not show association with EBF practice as opposed in studies done in other parts of Tanzania, Uganda or Norway [25, 26, 31, 43].

### Limitations of Study

Recall bias was a potential limitation due to the fact that information on EBF based on recall since birth and some women might not remember when they specifically introduced other liquids or solids. It is hoped by including women with children aged 6–12 months this may be minimized as recall period is shorter compared to using older children. Furthermore, information that was obtained relied on women’s self-report and women may over report the EBF practice due to social desirability bias and hence the observed prevalence of EBF may be overestimated. Socioeconomic and employment factors found in Muheza makes it difficult to generalize this study to Tanzania.

## Conclusions and Recommendations

Based on these findings, we concluded that the prevalence of EBF in Muheza district is 24.1 %, lower than the national prevalence and the coverage required for all infants to benefit from the advantages of EBF practice. These results show that knowledge on EBF is associated with EBF practice and prevalence.

Most (95 %) of mothers attended antenatal clinic during pregnancy, delivered at health facility, breastfed their children within 1 h after delivery and fed their children colostrum yet complementary feeding start early before the recommended period and this deprived children from getting full benefits of breastfeeding.

Using recall since birth for estimating EBF, it seems the prevalence of EBF is slowly rising in Tanzania. Shirima et al. [18, 22] papers showed that EBF was <2 % in rural and urban Morogoro, while Argnasson [21] showed it was 25 % in Tabora. Mgongo et al. [25] showed that using recall since birth the EBF prevalence was 20.9 % in Kilimanjaro and this study prevalence of 24 % and increase from earlier studies.

The following measures should be considered to improve EBF practice:

- Enhancing EBF counseling during ante and post natal period in order to improve EBF prevalence in the district. As was noted that knowledge on EBF is associated with EBF practice and prevalence. EBF counseling may be integrated in PMTCT counseling even if the mother is HIV negative.
- Refresher training for frontline health workers working in RCH and maternity wards on EBF, training to involve TBAs as they still serve pregnant and postpartum women.
- There should be plan to implement Baby-friendly Hospital Initiative (BFHI) and Infant and Young Child Feeding strategies in the district.
- Community based EBF education and support programs where possible to disseminate EBF awareness and knowledge and address the myths noted from the study.
